# Chloretone—The Ideal Local Anesthetic in Dental Surgery

**Published:** 1900-06-15

**Authors:** Michael Leo

**Affiliations:** New York City


### CHLORETONE—THE IDEAL LOCAL ANESTHETIC IN DENTAL SURGERY.

BY MICHAEL LEO, D D S., NEW YORK CITY.

Some time ago my attention was called to the superior advantages which chloretone possesses as a local anesthetic. I had formerly used cocain, but lately abandoned it in favor of beta-eucain I think cocain would have given satisfactory results if its toxic effect upon the heart had not so seriously inconvenienced my patients, and myself, as well. Indeed, I have been obliged to attend for hours upon persons who had been overcome by an injection of a solution of cocain.

I am firmly convinced that a simple operation, like the extraction of a tooth, does not justify the use of any drug that will give rise to such serious after-effects. Moreover, in many of my cases the use of cocain caused sloughing of the tissues, despite the usual antiseptic precautions.

After reading an article in the *Medical Record* for June 10, 1899, on the subject of local anesthetics, I experimented with beta-eucain in a large number of cases. This drug would have proved satisfactory, so far as its local anesthetic effect is concerned, but its injection caused considerable swelling in the surrounding tissues. Patients would return and ask me the most disagreeable questions as, for instance, "Doctor, are you sure your needle was clean?" "Have I not developed blood poisoning?" and so on. The swelling caused by beta-eucain is painless, and seems to be harmless; but people do not like to be disfigured, even if only for three or four days, for usually the swelling subsides within that period, and the tissues again assume a normal appearance

A pharmacist suggested to me that I make a trial of chloretone, a new local anesthetic now coming into use in the hospitals and clinics especially in minor surgery. Upon investigation I found that while chloretone is an efficient local anesthetic, it has no toxic effect upon the heart, and it does not cause sloughing or swelling after its use. Thereupon, I procured an ounce of the crystals and made two solutions according to directions. One solution, to be used in extracting, was prepared by mixing 15 percent of alcohol with 85 percent of distilled water, and adding enough chloretone to make a saturated solution. I have used this solution in hundreds of cases, with perfect success, and I am prepared to say, as a result of my experience, that chloretone possesses all the good qualities of cocain and beta-eucain, and does not cause any of the objectionable effects of either.

The second solution, which was made by mixing equal parts, by weight, of ether and chloretone, proved very efficient as an obtundent in preparing painful cavities for fillings, especially when sensitive dentin was being operated upon; also in setting crowns and in bridge work, which often gives rise to considerable pain, caused by the action of the glacial phosphoric acid with which the cement is mixed. This ethereal solution of chloretone should be employed by the careful practitioner when a live pulp must be removed. This can be done immediately and painlessly, after a thorough application of the solution.

I append brief notes of a few of my cases, for the benefit of the profession, and I hope that my experience will prove valuable to my confreres.

Mrs. S. B. Right upper third molar; pus sac. Injected 25 minims. Extraction after one minute. Three attempts were necessary, as the tooth was wedged and hard to extract. The patient experienced very little pain, even though it required almost three minutes to complete the operation.

W. S., aged eleven years. Right, lower, six-year molar. Two pus sacs. Fifty seconds after injection tooth extracted. Very little pain.

Mrs. L. Left, upper, second bicuspid; pus sac; chloretone injection, 20 minims; extraction after one minute. No pain at the time or afterward.

M. L. Left, lower, second molar; two pus sacs were painlessly lanced fifty seconds after injection. Extraction with slight pain. After-pain ceased within five minutes.

O. F. Left, upper, first molar; two pus sacs. The pain of the alveolar abscess was intense, but ceased fifteen seconds after injection. The tooth was extracted with very little pain, as the patient affirmed.

C. D. Left, upper third molar; pulp exposed; chloretone injection and painless extraction after fifty seconds.

A. R. Left, upper, second molar; alveolar abscess was lanced. Pain ceased after injection. Fifty seconds after painless extraction. Some months later this patient said the extraction was very neatly and painlessly done.

M. McG. Anterior root of left, lower first molar; pus sac. Forty seconds after injection, painless extraction. No after-pain so characteristic of alveolar abscess.

Miss E. L. Left, upper lateral incisor; right, upper lateral incisor. Injected 20 minims chloretone solution. One minute afterward painless extraction.

Mrs D. M. I injected the ethereal solution of chloretone into the pulp of a right, upper cuspid. As the needle of the syringe advanced into the pulp chamber, I pressed warm wax around it, thus closing the cavity so that the liquid could not escape. Forty seconds later I withdrew the instrument and wax, to permit the ether to evaporate. I also used the precaution to place the solution in a small test tube, which I held in my hand. Thus I was enabled to keep the temperature of the fluid at blood heat in order not to create pain by the injection of the cold solution.

I then removed the pulp with a broach one minute after the injection. The patient stated that the operation was painless.

Miss L. D. Right, second bicuspid. Right cuspid; 20 minims chlorelone solution; painless extraction fifty seconds later.

F. P. Right, lower cuspid; sensitive dentin. The mere touch of an instrument seemed to be unbearable. Ethereal solution of chloretone applied until white crystals deposited. I then proceeded to excavate. After a while it was necessary to repeat the application of chloretone, when I was enabled to complete my work without complaint from the patient.

M. M. Left, lower, six year molar; pulp exposure. Extraction, with very little pain, forty seconds after injection of chloretone solution.

M. D. Right, lower, first molar; exposed pulp. Chloretone injection; after forty seconds painless extraction.

F. P. Extraction left, lower, first molar; pus sac. Injection of 20 minims chloretone solution. In fifty seconds painless extraction. No after-pain.

M. R. Left, upper, third molar. Injection of 25 minims chloretone solution. After fifty seconds, painless extraction.

F. J. Right, lower, second molar; pus sac. Injected 20 minims chloretone solution. After fifty seconds, painless extraction. I might add that the pain of the abscess ceased at once after the chloretone had been injected.

F. P. Setting of gold crown, which was of necessity inserted deep under the gum in order to reach the edge of the root—a second, lower left bicuspid. I applied the ethereal solution of chloretone on a cotton pellet, until the white crystals were visible upon the gum. The crown was then set without pain. A few days before I became acquainted with the anesthetic properties of chloretone I placed a crown in a case similar to this one; the operation was very painful, on account of the action of the glacial phosphoric acid in the cement, it being necessary to use a very thin solution.—*Items of Interest.*

